# Supplementary figures and images for: Molecularly barcoded Zika virus libraries to probe in vivo evolutionary dynamics
Source: PLoS Pathog. 2018 Mar 28;14(3):e1006964. doi: 10.1371/journal.ppat.1006964 (PMC5891079; doi:10.1371/journal.ppat.1006964)

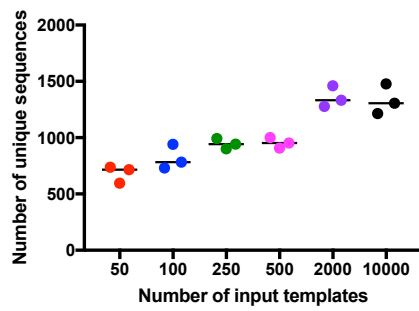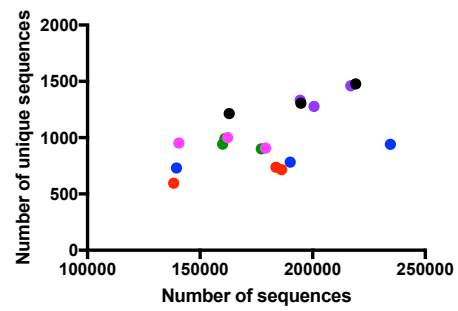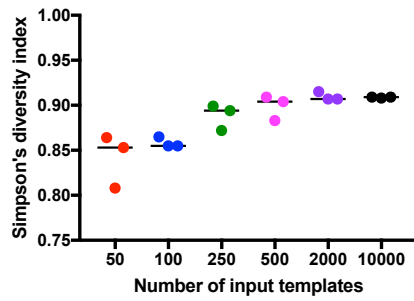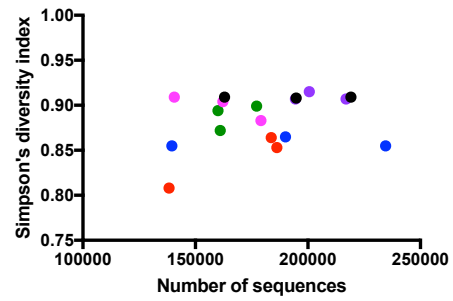

Supplement: S1 Fig — Sequence diversity vs. number of input templates (LHS) and total number of sequences per sample (RHS). The diversity measures include: number of unique sequences (upper) and Simpson’s diversity index (lower). The diversity for 3 replicate samples per input template number are shown. (PDF) [file ppat.1006964.s001.pdf]

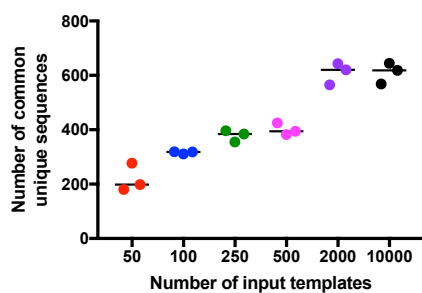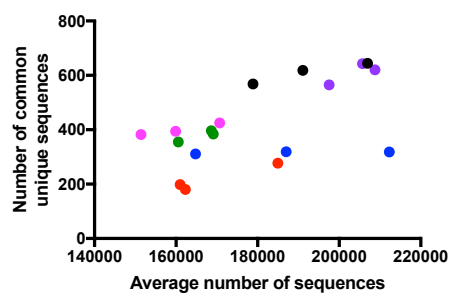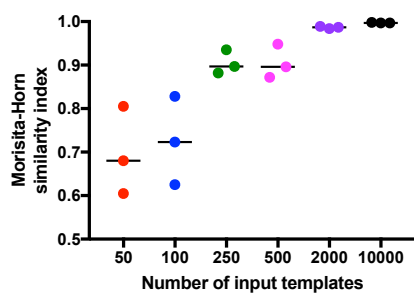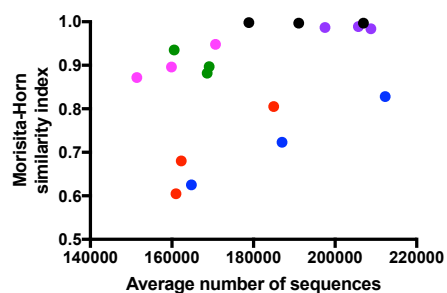

Supplement: S2 Fig — Similarity between pairs of replicate samples with the same input template number vs. number of input templates (LHS) and average total number of sequences (averaged between sample pairs) (RHS). The similarity measures include: number of common unique sequences (upper) and Morisita-Horn index (lower). The similarity between pairs of replicate samples per input template number are shown (i.e. RepA/RepB, RepA/RepC, and RepB/RepC). (PDF) [file ppat.1006964.s002.pdf]

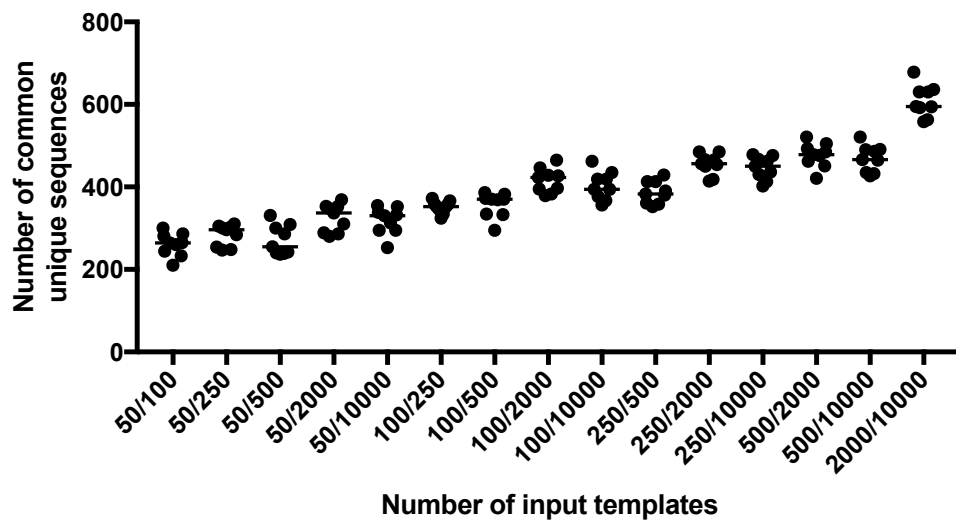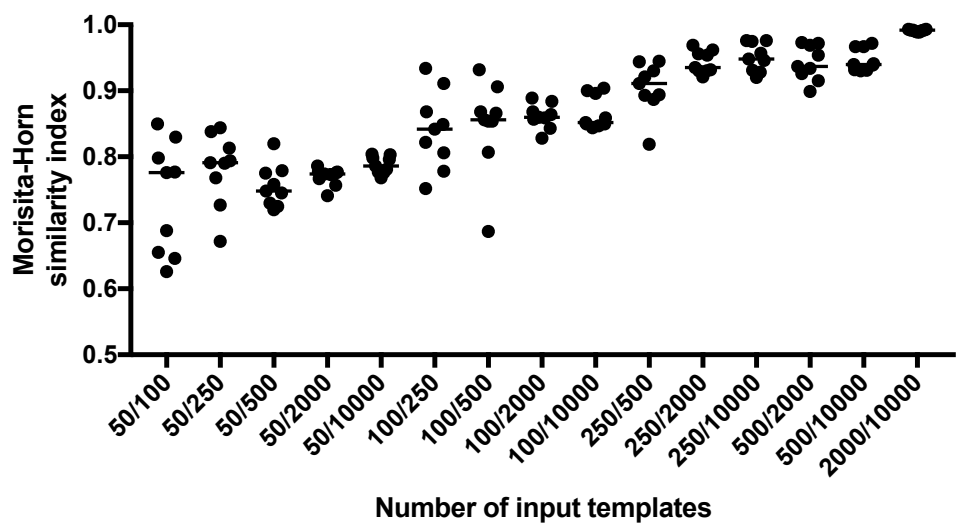

Supplement: S3 Fig — Similarity between pairs of replicate samples with different input template numbers vs. number of input templates for the pair (i.e. sample 1/sample 2). The similarity measures include: number of unique sequences common between samples (upper) and Morisita-Horn similarity index (lower). (PDF) [file ppat.1006964.s003.pdf]

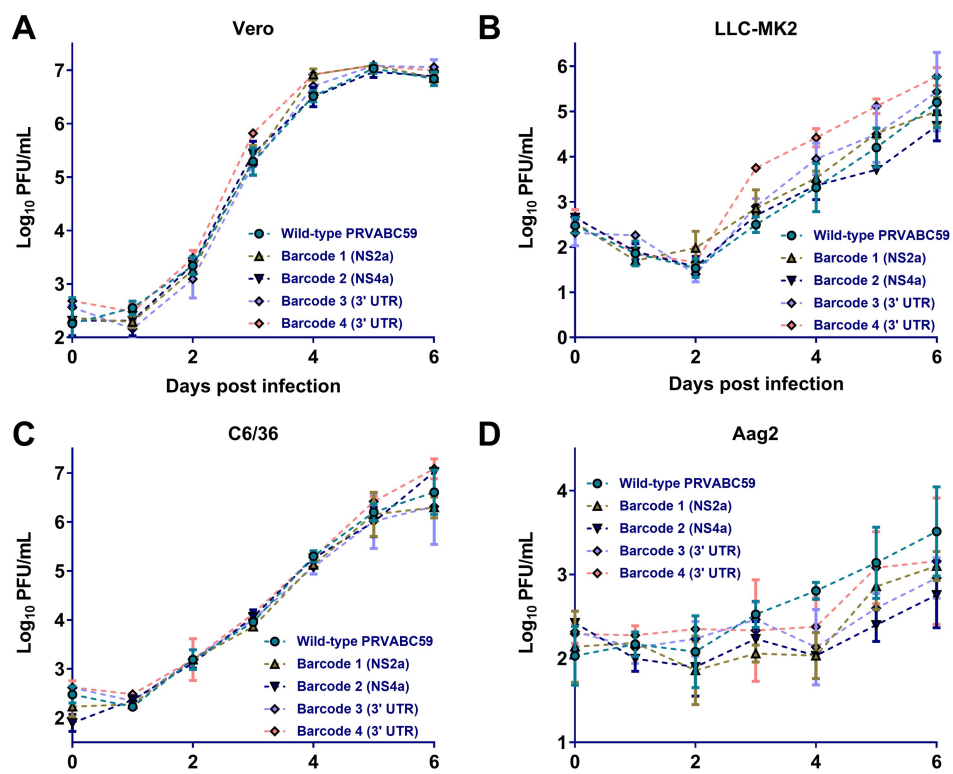

Supplement: S4 Fig — Cells were infected at an MOI of 0.01 PFU/cell. A.) Vero cells. B.) LLC-MK2 cells. C.) C6/36 cells. D.) Aag2 cells. For a full description see Weger-Lucarelli et al., manuscript submitted. (PDF) [file ppat.1006964.s004.pdf]
