# Supplementary material for: Molecularly barcoded Zika virus libraries to probe in vivo evolutionary dynamics
Source: PLoS Pathog. 2018 Mar 28;14(3):e1006964. doi: 10.1371/journal.ppat.1006964 (PMC5891079; doi:10.1371/journal.ppat.1006964)
Supplement: S1 Table — (DOCX) [file ppat.1006964.s005.docx]

**Table S1. Barcodes detected and their frequencies in the stock when using threshold A.**

| **Barcode name** | **Barcode Sequence** | **ZIKV BC-1.0 Stock_RepA** | **ZIKV BC-1.0 Stock_RepB** |
| --- | --- | --- | --- |
| BC_1 | CTCGCAGCACTGACTCCTCTTGCG | 21.15 | 21.36 |
| BC_2 | CTCGCTGCCCTCACACCTCTTGCA | 14.01 | 14.31 |
| BC_3 | CTGGCCGCGCTGACTCCTCTCGCT | 11.93 | 11.87 |
| BC_4 | CTGGCTGCACTAACTCCGCTGGCG | 6.97 | 6.81 |
| BC_5 | CTCGCTGCTCTGACTCCTCTCGCC | 5.17 | 5.01 |
| BC_6 | CTTGCAGCTCTAACCCCCCTAGCA | 5.16 | 4.42 |
| BC_7 | CTAGCCGCACTAACGCCGCTAGCC | 4.88 | 5.09 |
| BC_8 | CTGGCTGCACTGACTCCCCTAGCC | 4.37 | 4.31 |
| BC_9 | CTCGCGGCACTAACGCCGCTGGCG | 3.15 | 3.14 |
| BC_10 | CTAGCCGCCCTAACCCCGCTAGCG | 2.46 | 2.36 |
| BC_11 | CTGGCCGCGCTGACGCCGCTGGCG | 2.30 | 2.23 |
| BC_12 | CTTGCGGCCCTGACTCCTCTAGCG | 1.56 | 1.68 |
| BC_13 | CTCGCGGCGCTTACGCCTCTTGCC | 1.08 | 1.10 |
| BC_14 | CTTGCAGCGCTGACGCCTCTAGCC | 1.02 | 1.07 |
| BC_15 | CTAGCCGCTCTGACTCCGCTAGCG | 0.95 | 0.93 |
| BC_16 | CTTGCCGCTCTAACGCCCCTTGCC | 0.90 | 0.83 |
| BC_17 | CTCGCTGCCCTCACGCCGCTCGCT | 0.80 | 0.74 |
| BC_18 | CTAGCTGCTCTAACACCTCTAGCT | 0.61 | 0.56 |
| BC_19 | CTCGCAGCTCTCACGCCGCTGGCC | 0.59 | 0.58 |
| BC_20 | CTTGCCGCGCTTACACCTCTAGCC | 0.57 | 0.68 |
| BC_21 | CTTGCTGCCCTAACGCCGCTTGCT | 0.46 | 0.50 |
| BC_22 | CTGGCTGCTCTTACGCCACTCGCA | 0.37 | 0.37 |
| BC_23 | CTCGCGGCACTAACGCCCCTCGCA | 0.36 | 0.41 |
| BC_24 | CTGGCGGCACTAACGCCACTGGCT | 0.32 | 0.35 |
| BC_25 | CTAGCGGCGCTGACGCCACTCGCG | 0.32 | 0.33 |
| BC_26 | CTAGCTGCCCTCACTCCGCTCGCG | 0.30 | 0.39 |
| BC_27 | CTTGCGGCGCTAACGCCTCTTGCT | 0.30 | 0.35 |
| BC_28 | CTTGCTGCGCTCACTCCCCTCGCG | 0.28 | 0.29 |
| BC_29 | CTCGCAGCACTAACACCCCTAGCT | 0.27 | 0.25 |
| BC_30 | CTTGCCGCGCTCACGCCTCTTGCC | 0.27 | 0.28 |
| BC_31 | CTTGCCGCTCTGACTCCTCTTGCC | 0.23 | 0.16 |
| BC_32 | CTGGCCGCTCTGACCCCACTCGCA | 0.22 | 0.20 |
| BC_33 | CTAGCTGCTCTTACTCCTCTCGCG | 0.19 | 0.17 |
| BC_34 | CTAGCCGCCCTCACGCCTCTTGCC | 0.18 | 0.19 |
| BC_35 | CTTGCCGCGCTCACTCCACTTGCT | 0.18 | 0.14 |
| BC_36 | CTAGCAGCTCTCACTCCGCTTGCG | 0.17 | 0.23 |
| BC_37 | CTTGCTGCACTTACACCCCTTGCG | 0.15 | 0.17 |
| Zika_WT | CTGGCTGCTCTGACACCACTGGCC | 0.14 | 0.12 |
| BC_39 | CTGGCCGCGCTGACTCCTCTTGCG | 0.12 | 0.14 |
| BC_40 | CTCGCCGCCCTTACGCCACTTGCG | 0.11 | 0.12 |
| BC_41 | CTAGCTGCCCTTACCCCGCTGGCC | 0.11 | 0.09 |
| BC_42 | CTGGCCGCGCTGACTCCTCTCGCC | 0.10 | 0.12 |
| BC_43 | CTAGCTGCACTAACACCGCTTGCT | 0.10 | 0.10 |
| BC_44 | CTCGCCGCACTAACGCCGCTCGCA | 0.09 | 0.07 |
| BC_45 | CTAGCTGCTCTCACTCCTCTAGCA | 0.09 | 0.12 |
| BC_46 | CTCGCAGCACTGACTCCTCTCGCT | 0.08 | 0.07 |
| BC_47 | CTCGCGGCGCTTACACCCCTCGCG | 0.08 | 0.10 |
| BC_48 | CTCGCAGCACTGACTCCTCTTGCA | 0.07 | 0.08 |
| BC_49 | CTTGCGGCCCTAACACCCCTTGCC | 0.07 | 0.10 |
| BC_50 | CTCGCTGCCCTCACACCCCTTGCA | 0.06 | 0.03 |
| BC_51 | CTGGCTGCGCTAACTCCCCTCGCG | 0.06 | 0.08 |
| BC_52 | CTAGCCGCGCTTACGCCACTTGCT | 0.06 | 0.07 |
| BC_53 | CTTGCTGCGCTAACTCCCCTAGCG | 0.05 | 0.06 |
| BC_54 | CTTGCGGCACTTACCCCACTGGCT | 0.04 | 0.06 |
| BC_55 | CTCGCAGCACTGACTCCTCTTGCC | 0.03 | 0.06 |
| BC_56 | CTCGCTGCCCTCACACCTCTTGCG | 0.03 | 0.06 |
| BC_57 | CTTGCTGCACTGACACCACTAGCT | 0.01 | 0.08 |
| Other |  | 4.30 | 4.41 |
