# Supplementary material for: Molecularly barcoded Zika virus libraries to probe in vivo evolutionary dynamics
Source: PLoS Pathog. 2018 Mar 28;14(3):e1006964. doi: 10.1371/journal.ppat.1006964 (PMC5891079; doi:10.1371/journal.ppat.1006964)
Supplement: S2 Table — (DOCX) [file ppat.1006964.s006.docx]

**Table S2. Barcodes detected and their frequencies in the stock when using threshold B.**

| **Barcode name** | **Barcode Sequence** | **ZIKV BC-1.0 Stock_RepA** | **ZIKV BC-1.0 Stock_RepB** |
| --- | --- | --- | --- |
| BC_1 | CTCGCAGCACTGACTCCTCTTGCG | 21.15 | 21.36 |
| BC_2 | CTCGCTGCCCTCACACCTCTTGCA | 14.01 | 14.31 |
| BC_3 | CTGGCCGCGCTGACTCCTCTCGCT | 11.93 | 11.87 |
| BC_4 | CTGGCTGCACTAACTCCGCTGGCG | 6.97 | 6.81 |
| BC_5 | CTCGCTGCTCTGACTCCTCTCGCC | 5.17 | 5.01 |
| BC_6 | CTTGCAGCTCTAACCCCCCTAGCA | 5.16 | 4.42 |
| BC_7 | CTAGCCGCACTAACGCCGCTAGCC | 4.88 | 5.09 |
| BC_8 | CTGGCTGCACTGACTCCCCTAGCC | 4.37 | 4.31 |
| BC_9 | CTCGCGGCACTAACGCCGCTGGCG | 3.15 | 3.14 |
| BC_10 | CTAGCCGCCCTAACCCCGCTAGCG | 2.46 | 2.36 |
| BC_11 | CTGGCCGCGCTGACGCCGCTGGCG | 2.30 | 2.23 |
| BC_12 | CTTGCGGCCCTGACTCCTCTAGCG | 1.56 | 1.68 |
| BC_13 | CTCGCGGCGCTTACGCCTCTTGCC | 1.08 | 1.10 |
| BC_14 | CTTGCAGCGCTGACGCCTCTAGCC | 1.02 | 1.07 |
| BC_15 | CTAGCCGCTCTGACTCCGCTAGCG | 0.95 | 0.93 |
| BC_16 | CTTGCCGCTCTAACGCCCCTTGCC | 0.90 | 0.83 |
| BC_17 | CTCGCTGCCCTCACGCCGCTCGCT | 0.80 | 0.74 |
| BC_18 | CTAGCTGCTCTAACACCTCTAGCT | 0.61 | 0.56 |
| BC_19 | CTCGCAGCTCTCACGCCGCTGGCC | 0.59 | 0.58 |
| BC_20 | CTTGCCGCGCTTACACCTCTAGCC | 0.57 | 0.68 |
| BC_21 | CTTGCTGCCCTAACGCCGCTTGCT | 0.46 | 0.50 |
| BC_22 | CTGGCTGCTCTTACGCCACTCGCA | 0.37 | 0.37 |
| BC_23 | CTCGCGGCACTAACGCCCCTCGCA | 0.36 | 0.41 |
| BC_24 | CTAGCGGCGCTGACGCCACTCGCG | 0.32 | 0.33 |
| BC_25 | CTGGCGGCACTAACGCCACTGGCT | 0.32 | 0.35 |
| BC_26 | CTAGCTGCCCTCACTCCGCTCGCG | 0.30 | 0.39 |
| BC_27 | CTTGCGGCGCTAACGCCTCTTGCT | 0.30 | 0.35 |
| BC_28 | CTTGCTGCGCTCACTCCCCTCGCG | 0.28 | 0.29 |
| BC_29 | CTTGCCGCGCTCACGCCTCTTGCC | 0.27 | 0.28 |
| BC_30 | CTCGCAGCACTAACACCCCTAGCT | 0.27 | 0.25 |
| BC_31 | CTTGCCGCTCTGACTCCTCTTGCC | 0.23 | 0.16 |
| BC_32 | CTGGCCGCTCTGACCCCACTCGCA | 0.22 | 0.20 |
| BC_33 | CTAGCTGCTCTTACTCCTCTCGCG | 0.19 | 0.17 |
| BC_34 | CTTGCCGCGCTCACTCCACTTGCT | 0.18 | 0.14 |
| BC_35 | CTAGCCGCCCTCACGCCTCTTGCC | 0.18 | 0.19 |
| BC_36 | CTAGCAGCTCTCACTCCGCTTGCG | 0.17 | 0.23 |
| BC_37 | CTTGCTGCACTTACACCCCTTGCG | 0.15 | 0.17 |
| Zika_WT | CTGGCTGCTCTGACACCACTGGCC | 0.14 | 0.12 |
| BC_39 | CTGGCCGCGCTGACTCCTCTTGCG | 0.12 | 0.14 |
| Other |  | 5.54 | 5.88 |
