# Supplementary material for: Molecularly barcoded Zika virus libraries to probe in vivo evolutionary dynamics
Source: PLoS Pathog. 2018 Mar 28;14(3):e1006964. doi: 10.1371/journal.ppat.1006964 (PMC5891079; doi:10.1371/journal.ppat.1006964)
Supplement: S3 Table — (DOCX) [file ppat.1006964.s007.docx]

**Table S3. Barcodes detected and their frequencies in the stock when using threshold C.**

| **Barcode name** | **Barcode Sequence** | **ZIKV BC-1.0 Stock_RepA** | **ZIKV BC-1.0 Stock_RepB** |
| --- | --- | --- | --- |
| BC_1 | CTCGCAGCACTGACTCCTCTTGCG | 21.15 | 21.36 |
| BC_2 | CTCGCTGCCCTCACACCTCTTGCA | 14.01 | 14.31 |
| BC_3 | CTGGCCGCGCTGACTCCTCTCGCT | 11.93 | 11.87 |
| BC_4 | CTGGCTGCACTAACTCCGCTGGCG | 6.97 | 6.81 |
| BC_5 | CTCGCTGCTCTGACTCCTCTCGCC | 5.17 | 5.01 |
| BC_6 | CTTGCAGCTCTAACCCCCCTAGCA | 5.16 | 4.42 |
| BC_7 | CTAGCCGCACTAACGCCGCTAGCC | 4.88 | 5.09 |
| BC_8 | CTGGCTGCACTGACTCCCCTAGCC | 4.37 | 4.31 |
| BC_9 | CTCGCGGCACTAACGCCGCTGGCG | 3.15 | 3.14 |
| BC_10 | CTAGCCGCCCTAACCCCGCTAGCG | 2.46 | 2.36 |
| BC_11 | CTGGCCGCGCTGACGCCGCTGGCG | 2.30 | 2.23 |
| BC_12 | CTTGCGGCCCTGACTCCTCTAGCG | 1.56 | 1.68 |
| BC_13 | CTCGCGGCGCTTACGCCTCTTGCC | 1.08 | 1.10 |
| BC_14 | CTTGCAGCGCTGACGCCTCTAGCC | 1.02 | 1.07 |
| BC_15 | CTAGCCGCTCTGACTCCGCTAGCG | 0.95 | 0.93 |
| BC_16 | CTTGCCGCTCTAACGCCCCTTGCC | 0.90 | 0.83 |
| BC_17 | CTCGCTGCCCTCACGCCGCTCGCT | 0.80 | 0.74 |
| BC_18 | CTAGCTGCTCTAACACCTCTAGCT | 0.61 | 0.56 |
| BC_19 | CTCGCAGCTCTCACGCCGCTGGCC | 0.59 | 0.58 |
| BC_20 | CTTGCCGCGCTTACACCTCTAGCC | 0.57 | 0.68 |
| Zika_WT | CTGGCTGCTCTGACACCACTGGCC | 0.14 | 0.12 |
| Other |  | 10.23 | 10.80 |
