# Supplementary material for: Molecularly barcoded Zika virus libraries to probe in vivo evolutionary dynamics
Source: PLoS Pathog. 2018 Mar 28;14(3):e1006964. doi: 10.1371/journal.ppat.1006964 (PMC5891079; doi:10.1371/journal.ppat.1006964)
Supplement: S4 Table — (DOCX) [file ppat.1006964.s008.docx]

**Table** **S4.** Number of reads spanning barcode region that were interrogated when sequencing the titration of ZIKV-BC-1.0

| \| Sample \| Replicate \| Number of reads \| \| --- \| --- \| --- \| \| 50 copies \| A \| 186,244 \| \| B \| 138,322 \| \| C \| 183,671 \| \| 100 copies \| A \| 234,502 \| \| B \| 190,077 \| \| C \| 139,509 \| \| 250 copies \| A \| 160,060 \| \| B \| 161,021 \| \| C \| 177,246 \| \| 500 copies \| A \| 179,137 \| \| B \| 140,626 \| \| C \| 162,197 \| \| 2000 copies \| A \| 194,445 \| \| B \| 200,608 \| \| C \| 216,946 \| \| 10,000 copies \| A \| 162,997 \| \| B \| 194,760 \| \| C \| 219,146 \| |  |  |
| --- | --- | --- | --- | --- | --- | --- | --- | --- | --- | --- | --- | --- | --- | --- | --- | --- | --- | --- | --- | --- | --- | --- | --- | --- | --- | --- | --- | --- | --- | --- | --- | --- | --- | --- | --- | --- | --- | --- | --- | --- | --- | --- | --- | --- | --- | --- | --- |
