# Supplementary material for: Molecularly barcoded Zika virus libraries to probe in vivo evolutionary dynamics
Source: PLoS Pathog. 2018 Mar 28;14(3):e1006964. doi: 10.1371/journal.ppat.1006964 (PMC5891079; doi:10.1371/journal.ppat.1006964)
Supplement: S5 Table — (DOCX) [file ppat.1006964.s009.docx]

**Table S5. Number of viral templates used in the titration analysis of ZIKV-BC-1.0.**

| Sample | Number of vRNA templates put into the cDNA synthesis reaction | Number of theoretical cDNA templates put into the PCR reaction* |
| --- | --- | --- |
| 50 copies | 50 | 30 |
| 100 copies | 100 | 60 |
| 250 copies | 250 | 149 |
| 500 copies | 500 | 298 |
| 2000 copies | 2000 | 1,190 |
| 10,000 copies | 10,000 | 5,952 |

The number of cDNA templates was calculated based on the number of vRNA templates that were put into the cDNA synthesis reaction, and then the amount of cDNA that was used for the PCR reaction.
