# Supplementary material for: Molecularly barcoded Zika virus libraries to probe in vivo evolutionary dynamics
Source: PLoS Pathog. 2018 Mar 28;14(3):e1006964. doi: 10.1371/journal.ppat.1006964 (PMC5891079; doi:10.1371/journal.ppat.1006964)
Supplement: S6 Table — (DOCX) [file ppat.1006964.s010.docx]

**Table S6. ANOVA table.**

|  | Sum of Squares | df | Mean square | F | Significance |
| --- | --- | --- | --- | --- | --- |
| Between Groups | 70.59 | 2 | 35.3 | 0.887 | 0.460 |
| Within Groups | 238.7 | 6 | 39.78 |  |  |
| Total | 309.3 | 8 |  |  |  |
