# Supplementary material for: Molecularly barcoded Zika virus libraries to probe in vivo evolutionary dynamics
Source: PLoS Pathog. 2018 Mar 28;14(3):e1006964. doi: 10.1371/journal.ppat.1006964 (PMC5891079; doi:10.1371/journal.ppat.1006964)
Supplement: S7 Table — (DOCX) [file ppat.1006964.s011.docx]

**Table S7. Complete list of tissues examined for ZIKV-BC-1.0 RNA from 776301 and her fetus.**

|  | **Dam** | **Fetus** |
| --- | --- | --- |
| mesenteric LN | - | ND |
| spleen | - | ND |
| adipose tissue | ND | - |
| adrenal gland | ND | - |
| amniotic/chorionic membrane | ND | - |
| aorta-thoracic | ND | - |
| articular-cartilage | ND | - |
| axillary LN | ND | - |
| bile aspirate | ND | - |
| bone marrow | ND | - |
| cerebrum (9 sections) | ND | - |
| cervical spinal cord | ND | - |
| colon | ND | - |
| cord blood-serum | ND | - |
| cornea | ND | - |
| decidua | ND | - |
| dura mater | ND | - |
| epidermis/dermis abdomen | ND | - |
| esophagus | ND | - |
| eye-aqueous humor | ND | - |
| femur bone | ND | - |
| heart | ND | - |
| inguinal LN | ND | - |
| jejunum | ND | - |
| kidney | ND | - |
| liver | ND | - |
| lumbar spinal cord | ND | - |
| lung | ND | - |
| meconium | ND | - |
| mesenteric LN | ND | - |
| muscle-quadriceps | ND | - |
| optic nerve | ND | - |
| ovary | ND | - |
| pancreas | ND | - |
| pericardium | ND | - |
| pituitary gland | ND | - |
| placental disc 1 | ND | - |
| placental disc 2 | ND | 78 copies/mg |
| retina | ND | - |
| sclera | ND | - |
| spleen | ND | - |
| stomach | ND | - |
| submandibular LN | ND | - |
| terminal blood draw-plasma | ND | - |
| terminal CSF | ND | - |
| thoracic spinal cord | ND | - |
| thymus | ND | - |
| thyroid | ND | - |
| tongue | ND | - |
| tonsil | ND | - |
| tracheobroncial LN | ND | - |
| umbilical cord | ND | - |
| urinary bladder | ND | - |
| urine-aspirate | ND | - |
| uterus | ND | - |
| uterus-placental bed | ND | - |

-, ZIKV RNA below the limit of detection

LN, lymph node

ND, no data.
