# Supplementary material for: Molecularly barcoded Zika virus libraries to probe in vivo evolutionary dynamics
Source: PLoS Pathog. 2018 Mar 28;14(3):e1006964. doi: 10.1371/journal.ppat.1006964 (PMC5891079; doi:10.1371/journal.ppat.1006964)
Supplement: S8 Table — (DOCX) [file ppat.1006964.s012.docx]

| **Table S8.** Number of reads spanning barcode region that were interrogated when sequencing ZIKV-BC-1.0 and ZIKV-IC from nonpregnant animals.   \| Paper sample title \| Replicate \| # of reads \| \| --- \| --- \| --- \| \| ZIKV-BC-1.0 \| A \| 34,041 \| \| B \| 34,503 \| \| 715132 Day 3 \| A \| 12,010 \| \| B \| 88,841 \| \| 715132 Day 5 \| A \| 14,124 \| \| B \| 19,190 \| \| 688387 Day 3 \| A \| 31,841 \| \| B \| 60,600 \| \| 688387 Day 5 \| A \| 5,235 \| \| B \| 15,629 \| \| 514982 Day 2 \| A \| 30,063 \| \| B \| 62,957 \| \| ZIKV-IC \| A \| 38,893 \| \| B \| 30,267 \| \| 296168 Day 3 \| A \| 77,574 \| \| B \| 47,862 \| \| 296168 Day 5 \| A \| 16,647 \| \| B \| 18,353 \| \| 962498 Day 3 \| A \| 19,848 \| \| B \| 43,192 \| \| 962498 Day 5 \| A \| 23,329 \| \| B \| 17,917 \| \| 118693 Day 3 \| A \| 56,737 \| \| B \| 31,558 \| |
| --- | --- | --- | --- | --- | --- | --- | --- | --- | --- | --- | --- | --- | --- | --- | --- | --- | --- | --- | --- | --- | --- | --- | --- | --- | --- | --- | --- | --- | --- | --- | --- | --- | --- | --- | --- | --- | --- | --- | --- | --- | --- | --- | --- | --- | --- | --- | --- | --- | --- | --- | --- | --- | --- | --- | --- | --- | --- | --- | --- | --- | --- | --- | --- |
