# Supplementary material for: Molecularly barcoded Zika virus libraries to probe in vivo evolutionary dynamics
Source: PLoS Pathog. 2018 Mar 28;14(3):e1006964. doi: 10.1371/journal.ppat.1006964 (PMC5891079; doi:10.1371/journal.ppat.1006964)
Supplement: S9 Table — (DOCX) [file ppat.1006964.s013.docx]

| **Table S9.** Number of reads spanning barcode region that were interrogated when sequencing ZIKV-BC-1.0 from 776301.   \| Paper sample title \| Replicate \| # of reads \| \| --- \| --- \| --- \| \| ZIKV-BC-1.0 \| A \| 469,442 \| \| B \| 559,056 \| \| 776301 Day 3 \| A \| 597,349 \| \| B \| 544,054 \| \| 776301 Day 5 \| A \| 620,447 \| \| B \| 519,425 \| \| 776301 Day 7 \| A \| 127,414 \| \| B \| 106,991 \| \| 776301 Day 8 \| A \| 74,197 \| \| B \| 71,526 \| \| 776301 Day 10 \| A \| 99,852 \| \| B \| 67,989 \| \| 776301 Day 15 \| A \| 69,721 \| \| B \| 55,400 \| \| 776301 Day 18 \| A \| 427,830 \| \| B \| 353,683 \| \| 776301 Day 22 \| A \| 58,182 \| \| B \| 99,415 \| \| 776301 Day 25 \| A \| 259,398 \| \| B \| 138,265 \| \| 776301 Day 29 \| A \| 41,702 \| \| B \| 24,802 \| \| 776301 Day 32 \| A \| 289,547 \| \| B \| 332,621 \| \| 776301 Day 36 \| A \| 73,572 \| \| B \| 57,232 \| \| 776301 Day 39 \| A \| 198,958 \| \| B \| 306,826 \| \| 776301 Day 43 \| A \| 56,820 \| \| B \| 65,835 \| \| 776301 Day 46 \| A \| 146,484 \| \| B \| 224,969 \| \| 776301 Day 50 \| A \| 61,209 \| \| B \| 66,259 \| \| 776301 Day 57 \| A \| 61,388 \| \| B \| 18,291 \| \| 776301 Day 60 \| A \| 97,753 \| \| B \| 37,626 \| \| 776301 Day 67 \| A \| 37,517 \| \| B \| 34,951 \| |
| --- | --- | --- | --- | --- | --- | --- | --- | --- | --- | --- | --- | --- | --- | --- | --- | --- | --- | --- | --- | --- | --- | --- | --- | --- | --- | --- | --- | --- | --- | --- | --- | --- | --- | --- | --- | --- | --- | --- | --- | --- | --- | --- | --- | --- | --- | --- | --- | --- | --- | --- | --- | --- | --- | --- | --- | --- | --- | --- | --- | --- | --- | --- | --- | --- | --- | --- | --- | --- | --- | --- | --- | --- | --- | --- | --- | --- | --- | --- | --- | --- | --- | --- | --- | --- | --- | --- | --- | --- | --- | --- | --- | --- | --- | --- | --- | --- | --- | --- | --- | --- | --- | --- | --- |
