# Supplementary material for: Molecularly barcoded Zika virus libraries to probe in vivo evolutionary dynamics
Source: PLoS Pathog. 2018 Mar 28;14(3):e1006964. doi: 10.1371/journal.ppat.1006964 (PMC5891079; doi:10.1371/journal.ppat.1006964)
Supplement: S10 Table — (DOCX) [file ppat.1006964.s014.docx]

**Table S10.** Number of reads spanning barcode region that were interrogated when sequencing ZIKV-BC-1.0 from a mosquito who fed on 776301.

| Paper sample title | Replicate | # of reads |
| --- | --- | --- |
| Saliva | A | 90,702 |
|  | B | 94,276 |
| Body | A | 73,934 |
|  | B | 78,538 |
| Legs | A | 79,239 |
|  | B | 92,922 |
